# Real‐World Clinical Performance of a Novolimus‐Eluting Stent Versus a Sirolimus‐Eluting Stent

**DOI:** 10.1002/clc.24317

**Published:** 2024-07-02

**Authors:** Chun‐Chin Chang, Wei‐Ting Sung, Ya‐Wen Lu, Ming‐Ju Chuang, Yin‐Hao Lee, Yi‐Lin Tsai, Ruey‐Hsing Chou, Shao‐Sung Huang, Po‐Hsun Huang

**Affiliations:** ^1^ Department of Medicine, Division of Cardiology Taipei Veterans General Hospital Taipei Taiwan; ^2^ Cardiovascular Research Center National Yang Ming Chiao Tung University Taipei Taiwan; ^3^ Institute of Clinical Medicine National Yang Ming Chiao Tung University Taipei Taiwan; ^4^ Department of Medicine, Division of Cardiology Taichung Veterans General Hospital Taichung Taiwan; ^5^ Department of Medicine, Division of Cardiology Taipei City Hospital Yang Ming Branch Taipei Taiwan; ^6^ Department of Critical Care Medicine Taipei Veterans General Hospital Taipei Taiwan; ^7^ Healthcare and Services Center Taipei Veterans General Hospital Taipei Taiwan

**Keywords:** coronary artery disease, novolimus‐eluting stent, percutaneous coronary intervention, sirolimus‐eluting stent

## Abstract

**Introduction:**

The DESyne novolimus‐eluting coronary stent (NES) is a new‐generation drug‐eluting stent (DES) that is widely used, but clinical data are rarely reported for this stent. We compared the safety and effectiveness of the DESyne NES and the Orsiro bioresorbable polymer sirolimus‐eluting stent (SES) in patients undergoing percutaneous coronary intervention (PCI).

**Methods:**

This was a retrospective, single‐center, observational study. Between July 2017 and December 2022, patients who presented with chronic or acute coronary syndrome undergoing PCI with DESyne NES or Orsiro SES were consecutively enrolled in the present study. The primary endpoint, major adverse cardiovascular event (MACE), was a composite of cardiovascular death, target‐vessel myocardial infarction, or clinically driven target‐lesion revascularization.

**Results:**

A total of 776 patients (age 68.8 ± 12.2; 75.9% male) undergoing PCI were included. Overall, 231 patients with 313 lesions received NES and 545 patients with 846 lesions received SES. During a follow‐up duration of 784 ± 522 days, the primary endpoint occurred in 10 patients (4.3%) in the NES group and in 36 patients (6.6%) in the SES group. After multivariate adjustment, the risk of MACE did not significantly differ between groups (NES vs. SES, hazard ratio 0.74, 95% CI, 0.35–1.55, *p* = 0.425). The event rate of individual components of the primary endpoint was comparable between the two groups.

**Conclusions:**

Favorable and similar clinical outcomes were observed in patients undergoing PCI with either NES or SES in a medium‐term follow‐up duration. Future studies with adequately powered clinical endpoints are required for further evaluation.

## Graphical abstract

This study compares the safety and effectiveness of the DESyne novolimus‐eluting stent (NES) and the Orsiro bioresorbable polymer sirolimus‐eluting stent (SES) in patients undergoing percutaneous coronary intervention in a real‐world practice. The risk of MACE, a composite of cardiovascular death, target‐vessel myocardial infarction, or clinically driven target‐lesion revascularization, did not significantly differ between groups (NES vs. SES, hazard ratio 0.74, 95% CI, 0.35–1.55, *p* = 0.425). The event rate of individual components of the primary endpoint was comparable between the two groups.

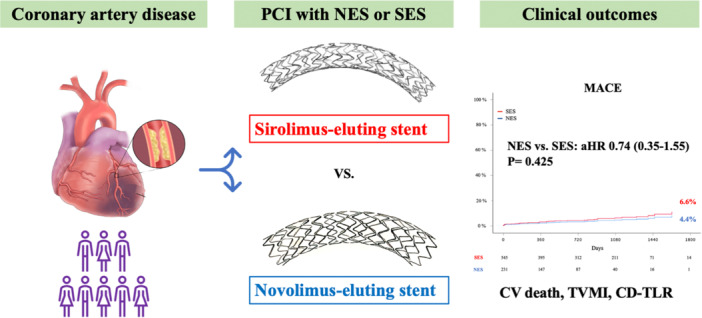

## 1 Introduction

The development of percutaneous coronary intervention (PCI) has led to improvements in the treatment of coronary artery disease (CAD) for more than four decades [1]. Evolution of drug‐eluting stent (DES) technologies has led to reduced adverse cardiovascular events in patients undergoing PCI [2]. Iteration of new‐generation DESs using thinner strut and drug coating, biocompatible or bioabsorbable polymers, and novel antiproliferative drugs has further improved the safety and efficacy of new‐generation DESs compared with old‐generation DESs. A meta‐analysis showed that ultrathin DESs (strut thickness < 70 μm) reduced the incidence of target‐lesion failure compared with contemporary thicker strut DESs [3].

The Orsiro sirolimus‐eluting stent (SES) is composed of a bioabsorbable polymer and ultrathin struts. A recent network meta‐analysis showed that the Orsiro SES was associated with a lower rate of target‐lesion failure at 1 year compared with other DESs [4].

The DESyne novolimus‐eluting stent (NES) utilizes novolimus, a metabolite of sirolimus that favorably requires a lower concentration of drug to inhibit neointimal proliferation, and was designed to reduce the rate of in‐stent restenosis. In the EXCELLA II study [5], the in‐stent late lumen loss was significantly lower in the NES compared to the zotarolimus‐eluting stent (ZES) at 9 months. Although the NES is increasingly being used in daily practice, the clinical performance of the NES is rarely reported after early studies and has not yet been tested in a randomized outcome trial.

In this context, we sought to investigate the safety and effectiveness of the NES compared to the SES in a real‐world practice.

## 2 Methods

### 2.1 Study Design and Participants

The present study was an observational, retrospective, single‐center study. From July 2017 to December 2022, patients presenting with chronic coronary syndrome or acute coronary syndrome (ACS) who underwent PCI using either DESyne NES (Elixir Medical Corporation, Sunnyvale, CA, USA) or Orsiri SES (BIOTRONIK, Buelach, Switzerland) at Taipei Veterans General Hospital were consecutively enrolled in the present study. The study was approved by the Taipei Veterans General Hospital's research ethics committee and was conducted in accordance with the Declaration of Helsinki. A waiver of documentation of patient informed consent was granted.

### 2.2 Devices

The DESyne NES is a CE‐marked DES consisting of a cobalt–chromium alloy with a strut thickness of 80 μm designed to optimize vessel coverage, flexibility, and deliverability [5]. The DESyne NES polymer is a durable poly *n*‐butyl methacrylate (PBMA) polymer with a coating thickness of < 3 μm. Novolimus is a macrocyclic lactone that demonstrates a high potency to inhibit human smooth muscle cells in in vitro studies. Novolimus inhibits the activation of the regulatory kinase mTOR. This inhibition suppresses cytokine‐driven cell proliferation, inhibiting progression from the G1 phase to the S phase of the cell cycle. This feature of novolimus allows a lower drug dose of 5 μg/mL of stent length.

The Orsiro SES consists of an ultrathin 60‐μm strut cobalt–chromium design with a bioresorbable, poly‐l‐lactic acid polymer coating that elutes the antiproliferative drug sirolimus (1.4 μg/mm^2^). This bioresorbable polymer SES has been shown to be noninferior to durable polymer everolimus‐eluting stents in randomized trials [6].

### 2.3 Procedures

PCI was performed according to standard clinical practice in the catheterization laboratory. Patients with stable CAD received dual antiplatelet therapy (DAPT) for at least 6 months after PCI, followed by aspirin monotherapy. Patients with ACS received DAPT for 12 months after PCI, followed by aspirin monotherapy. The duration of DAPT and the choice of P2Y12 inhibitors were at the discretion of the treating physicians according to patients' ischemic and bleeding risks. Patients were followed up by hospital visits every 3 months to assess clinical status and adverse events.

### 2.4 Clinical Endpoints

The primary endpoint of this study was major adverse cardiovascular event (MACE) during clinical follow‐up, defined as a composite of cardiovascular death, target‐vessel myocardial infarction (TVMI), or clinically driven target‐lesion revascularization (CD‐TLR). MI was defined according to the Fourth Universal Myocardial Infarction definition [7]. CD‐TLR and stent thrombosis were defined based on the Academic Research Consortium‐2 Consensus [8]. MACE was analyzed in a hierarchical manner.

### 2.5 Statistical Analysis

Categorical variables were presented as percentages and numbers. Continuous variables were presented as mean ± standard deviation. Baseline characteristics and procedural data were compared using Student's *t*‐test for continuous variables and the *χ* ^2^ test for categorical data. Survival curves were constructed using Kaplan–Meier estimates and the log‐rank test was used to compare between‐group differences. Univariate and multivariate Cox regression analyses were performed to determine risk factors that associated with MACE during the follow‐up.

Clinical variables for univariate analysis were selected based on the scientific literature. When the *p* value was less than 0.1 in the univariate analysis, the variable was included in the multivariate analysis, except for the type of stent used. A hazard ratio was reported with 95% confidence intervals based on the Cox regression model. A two‐sided *p* value of less than 0.05 was considered to indicate statistical significance. Data were analyzed using SPSS software (version 25, SPSS, Chicago, IL, USA).

## 3 Results

### 3.1 Patients; Baseline Characteristics and Follow‐Up

Between July 2017 and December 2022, a total of 776 patients (mean age 68.8 ± 12.2; 75.9% male) undergoing PCI were included in the study. Among them, 231 patients with 313 lesions received DESyne NES and 545 patients with 846 lesions received Orsiro SES, and the mean follow‐up duration was 637 ± 458 and 844 ± 535 days, respectively. Baseline characteristics were similar between groups (Table 1). Nevertheless, patients in the SES group more frequently had prior MI and prior CABG than the NES group.

**Table 1:** Baseline characteristics.

Baseline characteristics	All patients (*n* = 776)	NES (*n* = 231)	SES (*n* = 545)	*p* value
Age (years)	68.8 ± 12.2	69.8 ± 11.5	68.3 ± 12.4	0.111
Male	589 (75.9%)	172 (74.5%)	417 (76.5%)	0.582
Medical history				
Hypertension	576 (74.2%)	173 (74.9%)	403 (73.9%)	0.858
Diabetes mellitus	365 (47.0%)	100 (43.3%)	265 (48.6%)	0.182
Chronic kidney disease	216 (27.8%)	67 (29.0%)	149 (27.3%)	0.662
End‐stage renal disease	74 (9.5%)	28 (12.1%)	46 (8.4%)	0.141
Prior MI	99 (12.8%)	21 (9.1%)	78 (14.3%)	0.046
Prior PCI	230 (29.6%)	67 (29.0%)	163 (29.9%)	0.864
Prior CABG	22 (2.8%)	2 (0.9%)	20 (3.7%)	0.032
Prior stroke	88 (11.3%)	25 (10.8%)	63 (11.6%)	0.806
Heart failure	130 (16.8%)	38 (16.5%)	92 (16.9%)	0.917
Atrial fibrillation	54 (7.0%)	18 (7.8%)	36 (6.6%)	0.541
COPD	47 (6.1%)	16 (6.9%)	31 (5.7%)	0.513
PAD	62 (8.0%)	19 (8.2%)	43 (7.9%)	0.885
Clinical presentation				0.244
Stable CAD	518 (66.8%)	147 (63.6%)	371 (68.1%)	
Acute coronary syndrome	258 (33.2%)	84 (36.4%)	174 (31.9%)	
Unstable angina	81 (10.4%)	34 (14.7%)	47 (8.6%)	
NSTEMI	105 (13.5%)	34 (14.7%)	71 (13.0%)	
STEMI	72 (9.3%)	16 (6.9%)	56 (10.3%)	
Left main disease	107 (13.8%)	28 (12.1%)	79 (14.5%)	0.426
Number of diseased vessels				0.144
Single‐vessel disease	129 (16.5%)	47 (19.9%)	82 (15.1%)	
Two‐vessel disease	245 (31.6%)	82 (35.5%)	163 (29.9%)	
Three‐vessel disease	403 (51.9%)	103 (44.6%)	300 (55.0%)	
Medications				
Aspirin	713 (91.9%)	218 (94.4%)	495 (90.8%)	0.114
Clopidogrel	671 (86.5%)	206 (89.2%)	465 (85.3%)	0.169
Ticagrelor	64 (8.2%)	16 (6.9%)	48 (8.8%)	0.476
Prasugrel	18 (2.3%)	7 (3.0%)	11 (2.0%)	0.436
NOAC	44 (5.7%)	12 (5.2%)	32 (5.9%)	0.865

*Note:* Data were mean ± SD or number (%).

Abbreviations: CABG = coronary artery bypass graft; CAD = coronary artery disease; COPD = chronic obstructive pulmonary disease; MI = myocardial infarction; NSTEMI = non–ST elevation myocardial infarction; PAD = peripheral arterial disease; PCI = percutaneous coronary intervention; STEMI = ST elevation myocardial infarction.

### 3.2 Procedural Characteristics

Procedural characteristics are summarized in Table 2. Overall, transradial PCI was a favorable approach in both groups. Intravascular imaging was more frequently used in the NES group than the SES group (NES vs. SES: 48.1% vs. 34.7%, *p* < 0.001). The percentage of left main or proximal left anterior descending artery (LAD) disease was similar between groups. The number of stents used and the mean stent length were higher in the SES group. The mean diameter per stent was larger in the NES group.

**Table 2:** Procedural characteristics.

Procedure characteristics	All (776 patients) (1529/1162 lesions)	NES (231 patients) (434/313 lesions)	SES (545 patients) (1095/849 lesions)	*p* value
Vascular access				0.355
Radial	638 (82.2%)	195 (84.0%)	443 (81.3%)	
Femoral	138 (17.8%)	36 (16.0%)	102 (18.7%)	
Imaging‐guided PCI	300 (38.7%)	111 (48.1%)	189 (34.7%)	< 0.001
Angiography‐guided PCI	476 (61.3%)	120 (51.9%)	356 (65.3%)	
Locations of target lesions				
Patient level				
LM or proximal LAD	258 (33.2%)	78 (33.8%)	180 (33.0%)	0.867
Bifurcation involved	32 (4.1%)	8 (3.5%)	24 (4.4%)	0.363
Heavy calcification	57 (7.3%)	20 (8.7%)	37 (6.8%)	0.832
Rotational atherectomy	12 (1.5%)	6 (2.6%)	6 (1.1%)	0.122
Lesion level				
LM	57 (5.0%)	8 (2.6%)	49 (5.8%)	0.012
LAD	467 (40.2%)	151 (48.2%)	316 (37.2%)	
LCX	263 (22.6%)	72 (23.0%)	191 (22.5%)	
RCA	370 (31.8%)	82 (26.2%)	288 (33.9%)	
Bypass graft	5 (0.4%)	0 (0.0%)	5 (0.6%)	
Lesion classification				0.107
A	153 (13.2%)	49 (15.7%)	104 (12.2%)	
B1/B2	662 (57.0%)	183 (58.5%)	479 (56.4%)	
C	347 (29.8%)	81 (25.8%)	266 (31.3%)	
Number of study stents implanted	1.49 ± 0.78	1.35 ± 0.64	1.55 ± 0.83	0.001
Mean total study stent length (mm)	43.3 ± 25.5	36.2 ± 19.6	46.0 ± 27.0	< 0.001
Mean stent size (mm)	2.9 ± 0.4	3.0 ± 0.4	2.8 ± 0.4	< 0.001

*Note:* Data were mean ± SD or number (%).

Abbreviations: LAD = left anterior descending artery; LCX = left circumflex; LM = left main; NES = novolimus‐eluting stent; PCI = percutaneous coronary intervention; RCA = right coronary artery; SES = sirolimus‐eluting stent.

### 3.3 Clinical Outcomes

**Figure 1:**
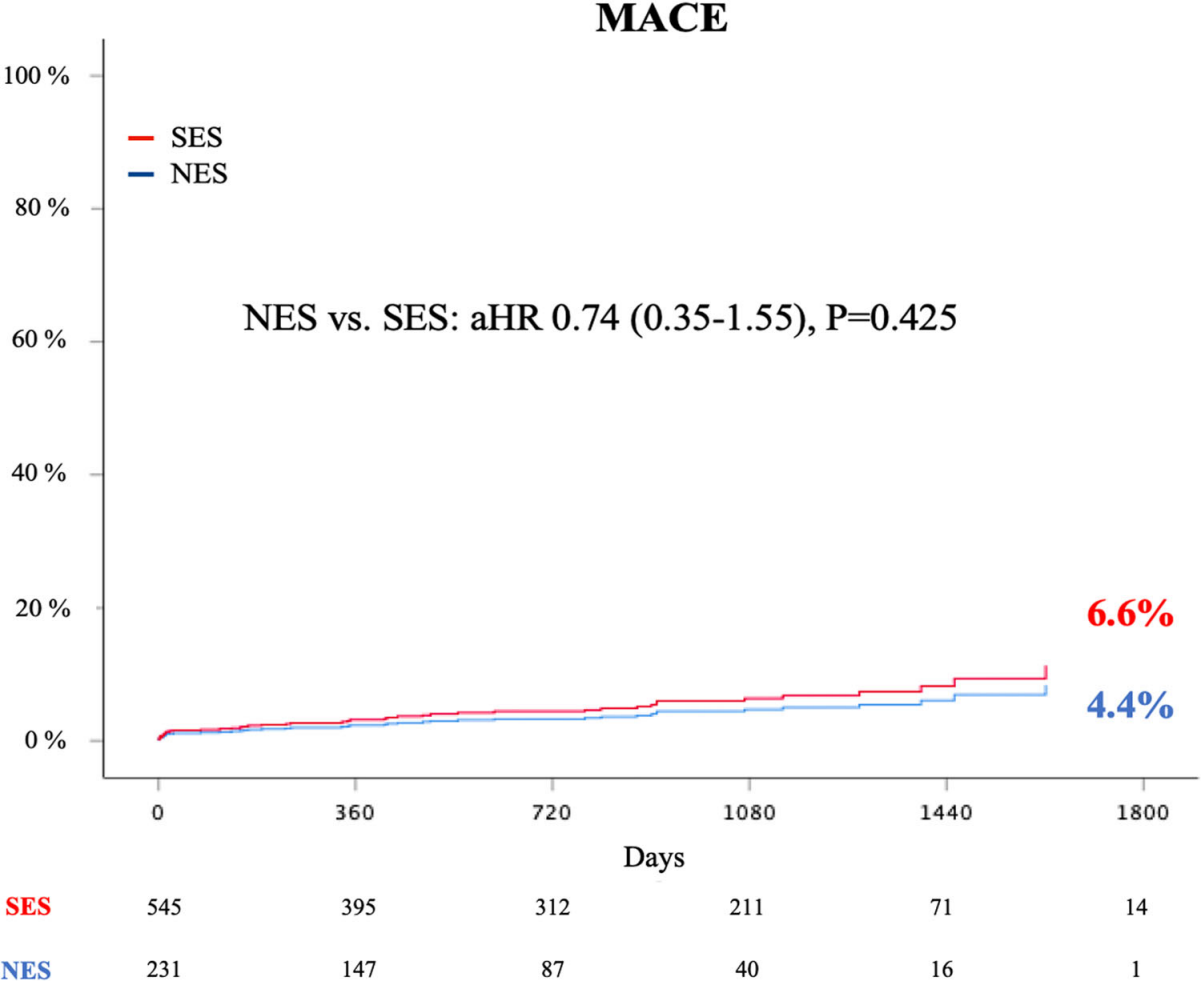
Cumulative MACE rates during follow‐up.

The primary endpoint of MACE occurred in 10 patients (4.3%) in the NES group and in 36 patients (6.6%) in the SES group during a mean follow‐up duration of 784 ± 522 days. Cox proportional hazards regression analysis was performed to explore the predictive factors of MACE (Table 3). In the univariate analysis, chronic kidney disease, left main or proximal LAD disease, and stent length were significantly associated with the occurrence of MACE. After multivariate adjustment, CKD and ACS were significantly associated with MACE, instead of the stent type (NES vs. SES: adjusted hazard ratio = 0.74, 95% CI, 0.35–1.55, *p* = 0.425) (Figure 1). Occurrences of cardiac death (3 [1.3%] vs. 18 [3.3%], *p* = 0.173), TVMI (0 [0.0%] vs. 5 [0.9%], *p* = 0.413), and CD‐TLR (7 [3.0%] vs. 19 [3.5%], *p* = 0.681) were similar for both stent types (Table 4). Definite or probable stent thrombosis rates were low in both groups (1 [0.43%] vs. 2 [0.36%], *p* = 1.000). The treatment effect was consistent across all subgroups (Supporting Information S1: Figure 1).

**Table 3:** Univariate and multivariate analyses for MACE.

Variables	Univariate analysis, hazard ratio (95% CI)	*p* value	Multivariate analysis, hazard ratio (95% CI)	*p* value
Age	1.02 (0.99–1.04)	0.220		
Man	0.75 (0.39–1.42)	0.378		
HTN	0.90 (0.47–1.70)	0.735		
DM	0.98 (0.55–1.75)	0.937		
Chronic kidney disease	3.05 (1.71–5.44)	< 0.001	2.82 (1.55–5.13)	< 0.001
Previous MI	1.32 (0.62–2.83)	0.477		
Previous PCI	0.80 (0.41–1.54)	0.498		
Previous CABG	2.16 (0.67–6.97)	0.198		
Previous stroke	1.14 (0.48–2.69)	0.767		
COPD	1.49 (0.53–4.15)	0.448		
Atrial fibrillation	2.21 (0.94–5.21)	0.071	1.74 (0.72–4.23)	0.218
PAD	2.06 (0.92–4.60)	0.080	2.02 (0.88–4.64)	0.096
ACS	1.65 (0.92–2.97)	0.091	1.82 (1.00–3.29)	0.049
LM or proximal LAD disease	1.84 (1.03–3.29)	0.040	1.56 (0.83–2.97)	0.170
Multivessel disease	2.12 (0.76–5.92)	0.150		
Image‐guided PCI	1.68 (0.93–3.01)	0.083	1.41 (0.74–2.70)	0.294
Number of study stents implanted	1.29 (0.97–1.73)	0.082	1.22 (0.58–2.56)	0.601
Mean stent size	1.84 (0.96–3.51)	0.065	1.86 (0.92–3.79)	0.086
Mean stent length	1.01 (1.00–1.02)	0.042	1.00 (0.98–1.03)	0.961
NES vs. SES	0.81 (0.40–1.65)	0.565	0.74 (0.35–1.55)	0.425

Abbreviations: ACS = acute coronary syndrome; CABG = coronary artery bypass graft; COPD = chronic obstructive pulmonary disease; LAD = left anterior descending artery; LM = left main; MI = myocardial infarction; NES = novolimus‐eluting stent; NSTEMI = non–ST elevation myocardial infarction; PAD = peripheral arterial disease; PCI = percutaneous coronary intervention; SES = sirolimus‐eluting stent; STEMI = ST elevation myocardial infarction.

**Table 4:** Comparison of clinical outcomes between NES and SES.

	All (*n* = 776)	NES (*n* = 231)	SES (*n* = 545)	HR (95% CI)	*p* value	HR^a^ (95% CI)	*p* value
MACE^b^	46 (5.9%)	10 (4.3%)	36 (6.6%)	0.81 (0.40–1.65)	0.565	0.74 (0.35–1.55)	0.425
Cardiac death	21 (2.7%)	3 (1.3%)	18 (3.3%)	0.43 (0.13–1.45)	0.173	0.45 (0.13–1.61)	0.221
TVMI	5 (0.6%)	0 (0.0%)	5 (0.9%)				
CD‐TLR	26 (3.4%)	7 (3.0%)	19 (3.5%)	1.20 (0.50–2.88)	0.681	0.99 (0.39–2.49)	0.980
All‐cause death	64 (8.2%)	20 (8.7%)	44 (8.1%)	1.31 (0.77–2.22)	0.326	1.30 (0.74–2.29)	0.356

Abbreviations: CD‐TLR = clinically driven target‐lesion revascularization; HR = hazard ratio; MACE = major adverse cardiovascular event; NES = novolimus‐eluting stent; SES = sirolimus‐eluting stent; TVMI = target‐vessel myocardial infarction.

^a^ Adjusted for chronic kidney disease, atrial fibrillation, peripheral arterial disease, left main or proximal LAD disease, acute coronary syndrome, the use of intracoronary imaging, number of stents implanted, and mean stent size and length.

^b^ MACE: a composite of cardiac death, TVMI, and CD‐TLR.

## 4 Discussion

The main findings of the present study were that the performance of the DESyne NES in terms of MACE at 2 years was comparable to the Orsiro SES. CKD was an independent predictor of MACE. The DESyne NES, in line with current‐generation DESs, was designed to overcome the limitation of second‐generation DESs, which have been reported to have a 2%–3% annual increase in the rate of the device‐oriented composite endpoint (DOCE) after 1 year [9]. Novolimus, a macrocyclic lactone with antiproliferative properties, has a similar efficacy to currently available antiproliferative drugs; however, it requires a lower dose and a thinner polymer coating. The EXCELLA II study had previously demonstrated both noninferiority and superiority of DESyne NES compared to Endeavor ZES for the primary endpoint of in‐stent late lumen loss at 9 months [5]. At the 5‐year follow‐up, the incidence of device‐oriented events was significantly lower in the DESyne NES group that in the control Endeavor ZES group in the EXCELLA II randomized clinical trial, suggesting superior efficacy and safety of the DESyne NES [10]. DOCEs for the DESyne NES were relatively low over 5 years, whereas those for the Endeavor ZES increased yearly (NES vs. ZES: 7.9% vs. 19.7%). At 5 years, TLR rates were also lower in favor of the DESyne NES compared with the Endeavor ZES (2.2% vs. 8.5%; *p* = 0.06). Nevertheless, the above‐mentioned study was not adequately powered to compare DOCE rates between the two groups. It is noteworthy that in contemporary PCI trials comparing a new stent platform to the current standard of care, a noninferiority study design is frequently used. However, with improvements in stent technology and PCI techniques, the event rate is decreasing and relatively low and therefore a large sample size is essential for an adequately powered randomized head‐to‐head study. However, a large‐scale randomized trial is costly and may not be fully applicable to the clinical setting. As such, there is a need for real‐world evidence to confirm the safety and efficacy of a new stent platform.

In our study, both the MACE rate and the TLR rate were low in the Orsiro SES group, which was in line with data from the randomized SORT OUT IX trial [11]. The performance of the Orsiro SES has been extensively investigated in several large‐scale randomized studies [6, 12, 13], whereas clinical data of the DESyne NES are scarce after the approval of CE certification. A DESyne NES postmarket follow‐up study is ongoing (NCT04375085). Therefore, our real‐world data provided evidence in terms of the performance of the DESyne NES in a real‐world practice.

Due to the advancement of stent technology, the current DES hardly demonstrates significant differences in terms of DOCE as compared to each other. Therefore, the baseline characteristics of patients may play a more important role in influencing long‐term outcomes. CKD has been known to be associated with adverse outcomes in patients with existing cardiovascular disease including increased mortality after PCI [14, 15]. In our study, the presence of CKD and ACS was more related to the occurrence of MACE, instead of the stent type.

### 4.1 Study Limitation

There were several study limitations that should be acknowledged. First, this is a retrospective, observational, single‐center study. Potential selection bias could not be fully avoided. The differences in baseline and procedural characteristics could not be entirely adjusted. Second, the present study does not have adequate power for the primary outcome. In this context, the study result is mainly hypothesis‐generating and should be interpreted with caution. Lastly, nonstudy stents could be used for nontarget lesions in our study. Even though the study endpoints were reported as target‐lesion– and target‐vessel–oriented outcomes, potential bias may exist.

## 5 Conclusion

Favorable and similar clinical outcomes were observed in patients undergoing PCI with either the DESyne NES or the Orsiro SES during a medium‐term follow‐up. Future large‐scale studies with adequately powered clinical endpoints are required for further evaluation.

## Conflicts of Interest

The authors declare no conflicts of interest.

## Supporting information

Supporting information.

## Data Availability

Research data will not be shared publicly.
