# Supplementary material for: Real‐World Clinical Performance of a Novolimus‐Eluting Stent Versus a Sirolimus‐Eluting Stent
Source: Clin Cardiol. 2024 Jul 2;47(7):e24317. doi: 10.1002/clc.24317 (PMC11217985; doi:10.1002/clc.24317)
Supplement: Supplementary file 1 — Supporting information. [file CLC-47-e24317-s001.docx]

**Real-World Clinical Performance of Novolimus-Eluting Stent versus Sirolimus-Eluting Stent**

**Authors**

Chun-Chin Chang^1,2,3^, Wei-Ting Sung^1,2^, Ya-Wen Lu^3,4^, Ming-Ju Chuang^1,2^, Yin-Hao Lee ^2,5^, Yi-Lin Tsai^1,2^, Ruey-Hsing Chou^1,2,3,6^, Shao-Sung Huang^1,2,7^, Po-Hsun Huang^1,2,3^

**Affiliations**

1. Division of Cardiology, Department of Medicine, Taipei Veterans General Hospital, Taipei, Taiwan.
2. Cardiovascular Research Center, National Yang Ming Chiao Tung University, Taipei, Taiwan.
3. Institute of Clinical Medicine, National Yang Ming Chiao Tung University, Taipei, Taiwan.
4. Division of Cardiology, Department of Medicine, Taichung Veterans General Hospital, Taichung, Taiwan.
5. Division of Cardiology, Department of Medicine, Taipei City Hospital, Yang Ming Branch, Taipei, Taiwan.
6. Department of Critical Care Medicine, Taipei Veterans General Hospital, Taipei, Taiwan.
7. Healthcare and Services Center, Taipei Veterans General Hospital, Taipei, Taiwan.

**Corresponding author:**

Chun-Chin Chang, MD, PhD

Division of cardiology, Department of Medicine

Taipei Veterans General Hospital, Taipei, Taiwan.

112, No. 201, Sec. 2, Shih-Pai Road, Taipei, Taiwan.

E-mail: [ccchang16@vghtpe.gov.tw](mailto:ccchang16@vghtpe.gov.tw)

&

Professor Po-Hsun Huang, MD, PhD

Division of cardiology, Department of Medicine

Taipei Veterans General Hospital, Taipei, Taiwan.

112, No. 201, Sec. 2, Shih-Pai Road, Taipei, Taiwan.

E-mail: [hunagbsvgh@gmail.com](mailto:hunagbsvgh@gmail.com)

**Supplementary Figure 1: Subgroups analysis**

**
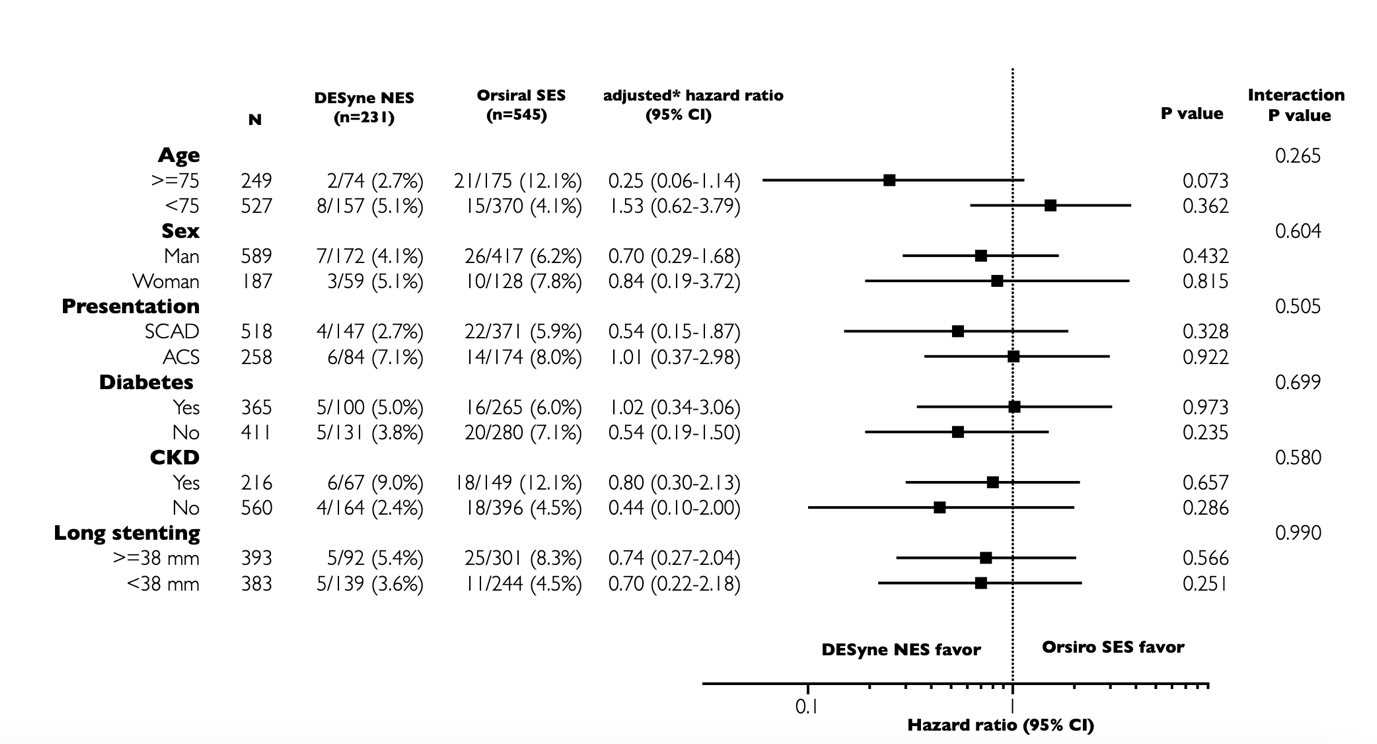
**

*Adjusted for chronic kidney disease, atrial fibrillation, peripheral arterial disease, left main or proximal LAD disease, acute coronary syndrome, the use of intracoronary imaging, number of stents implanted, mean stent size and length.
